# Alterations of Vaginal Microbiota in Women With Infertility and *Chlamydia trachomatis* Infection

**DOI:** 10.3389/fcimb.2021.698840

**Published:** 2021-08-03

**Authors:** Hongliang Chen, Li Wang, Lanhua Zhao, Lipei Luo, Shuling Min, Yating Wen, Wenbo Lei, Mingyi Shu, Zhongyu Li

**Affiliations:** ^1^Institute of Pathogenic Biology, Hengyang Medical College, Hunan Provincial Key Laboratory for Special Pathogens Prevention and Control, Hunan Province Cooperative Innovation Center for Molecular Target New Drug Study, University of South China, Hengyang, China; ^2^Chenzhou No.1 People’s Hospital, The First School of Clinical Medicine, Southern Medical University, Chenzhou, China

**Keywords:** *Chlamydia trachomatis*, infertile women, vaginal microbiota, 16S rRNA gene sequencing, *Lactobacillus*

## Abstract

*Chlamydia trachomatis* (*C. trachomatis*) is the most common etiological agent of bacterial sexually transmitted infections (STIs) worldwide and causes serious health sequelae such as cervicitis, pelvic inflammatory disease, and even infertility if ascending from the lower to the upper female genital tract. Previous studies have revealed the pivotal role of vaginal microbiota in susceptibility to STIs. However, alterations in the vaginal microbiota in women who are infertile and infected with *C. trachomatis* remain unknown. This study used metagenomic analysis of sequenced 16S rRNA gene amplicons to examine the vaginal microbial profiles of women with tubal infertility who were *C. trachomatis*-negative and those who were *C. trachomatis*-positive pre- and post-antibiotic treatment. Women who were *C. trachomatis*-negative and deemed healthy were recruited as references of eubiosis and dysbiosis. Women with tubal infertility and *C. trachomatis* infection presented a unique *Lactobacillus iners*-dominated vaginal microbiota rather than one dominated by *Lactobacillus crispatus* and displayed a decrease in *Lactobacillus*, *Bifidobacterium*, *Enterobacter*, *Atopobium*, and *Streptococcus*, accompanied by decreased levels of cytokines such as interferon (IFN)-γ and interleukin (IL)-10. This altered vaginal microbiota could be restored with varying degrees after standard treatment for *C. trachomatis*. This shift could be a predictive vaginal microbiota signature for *C. trachomatis* infection among females with tubal infertility, while no significant differences in phylum, class, and operational taxonomic unit (OTU) levels were observed between women with tubal infertility who were *C. trachomatis*-negative and healthy controls. This is the first study to provide data on the association of vaginal microbiota with *C. trachomatis* infection among women with tubal infertility and highlights unprecedented potential opportunities to predict *C. trachomatis* infection.

## Introduction

*Chlamydia trachomatis* (*C. trachomatis*) is an obligate intracellular parasitic bacterium that can infect both genital and non-genital sites including the cervix, rectum, and eyes (Mishori et al., 2012; Morrison et al., 2020). Genital *C. trachomatis* infection is a leading cause of bacterial sexually transmitted disease, responsible for more than 131 million emerging infections worldwide, and may manifest as mucopurulent cervicitis with a watery or purulent discharge and easily induced bleeding with a swab (Wiesenfeld, 2017). More than 60%–80% of infected women remains asymptomatic, which facilitates the spread of the pathogen and may lead to the development of a chronic infection (Woodhall et al., 2018). Furthermore, untreated *C. trachomatis* infection during labor can be vertically transmitted, resulting in conjunctivitis and pneumonitis in infants (Manavi, 2006).

*C. trachomatis* has a biphasic life cycle comprising a metabolically active noninfectious reticulate body (RB) and an infectious environmentally resistant elementary body (EB). The RB replicates by binary fission within the confines of the inclusion and differentiates into EBs at the end of the infectious replication cycle, while the EBs are closely followed by releasing from the cell to initiate new infection *via* cytolysis or endocytosis (Morrison, 2003; Chumduri et al., 2013). Various factors such as antibiotic treatment, host immunological response, or nutrient starvation disturb the *C. trachomatis* developmental cycle, and under such conditions, the EBs can convert to enlarged noninfectious aberrant bodies (ABs). This so-called “viable but non-cultivable growth stage” is associated with chronic and repeat infections that can lead to serious complications in women, including obstructive infertility, ectopic pregnancy, and preterm birth (den Hartog et al., 2006; Ziklo et al., 2016). Besides, persistent *C. trachomatis* infection enhanced the expression of *C. trachomatis* Hsp60 (cHsp60), capable of activating mononuclear cells or monocyte-derived macrophages producing E-selectin, intercellular adhesion molecule (ICAM)-1, and vascular cell adhesion molecule (VCAM)-1 and amplifying the ongoing inflammatory process by secreting pro-inflammatory cytokines (Kol et al., 1999; Contini and Seraceni, 2012). Moreover, this infection could also provoke the release of human heat shock protein (HSP)60, very similar to chlamydial heat shock protein (cHsp)60, which was firstly produced by early-stage embryos. In this regard, cross-reactive cHsp60 peptides elicited an immune response that can recognize the human hsp60 and increase the pathogenesis of genital chlamydial infection (Pockley, 2003; Witkin et al., 2017).

The female genital tract harbors a plethora of microorganisms that play an emerging role in human health and are influenced by host lifestyle, antibiotic use, and hormonal contraception (Ziklo et al., 2016). *Lactobacillus*-dominated vaginal microbiota is considered a marker of health status for healthy women (Ma et al., 2012; Pekmezovic et al., 2019), owing to its ability to produce lactic acid and multiple bacteriostatic and bactericidal compounds to protect against extraneous pathogenic bacteria (Valenti et al., 2018; Tamarelle et al., 2019). Women infected with *C. trachomatis* were hypothesized to undergo an alteration in their vaginal microbiota dominated by *Lactobacillus iners* or by diverse anaerobic bacteria (van der Veer et al., 2017).

Previous studies have revealed that *C. trachomatis* and human papillomavirus (HPV) might serve as mutual risk factors to increase the risk of infections (Seraceni et al., 2014; Chen et al., 2020). Consequently, we hypothesize that some vaginal microbiota may also accelerate or inhibit *C. trachomatis*-induced infertility among females. There are currently no data regarding alterations in the vaginal microbiota in women with tubal infertility who are infected with *C. trachomatis*. Therefore, the present study aimed to use 16S rRNA gene amplicon-based metagenomic analysis to characterize vaginal microbiota diversity in healthy women who were *C. trachomatis*-negative and receiving an annual physical examination, women with tubal infertility who were *C. trachomatis*-negative, and women with tubal infertility who were *C. trachomatis*-positive pre- and post-antibiotic treatment. Deciphering vaginal microbiota alterations induced by *C. trachomatis* in women with infertility will enhance the understanding of the potential interaction(s) of distinct bacterial communities with *C. trachomatis* infection.

## Materials and Methods

### Ethics Statement

All participants provided their written informed consent for participation in this study. The study was conducted in accordance with the Declaration of Helsinki, and the protocol was approved by the Ethics Committee of Chenzhou No. 1 People’s Hospital (CZ/1128).

### Study Design

From January 2019 to January 2020, women diagnosed as tubal infertile plus *C. trachomatis*-negative (CT-N) or -positive (CT-P) and seeking assistance at the assisted reproductive technology center (ART) and women who were *C. trachomatis*-negative (CT-C) and receiving an annual physical examination in the physical examination center (PEC) of a teaching hospital in Chenzhou were enrolled in the study. Participants with tubal infertility who were *C. trachomatis*-positive had study visits scheduled 60 days after standard treatment with azithromycin (CT-PT), a frontline antibiotic used to treat *C. trachomatis* infection. Standard treatment is 1 g azithromycin in a single oral dose according to *C. trachomatis* treatment guidelines approved by the Centers for Disease Control and Prevention (CDC) (Workowski et al., 2015).

All enrolled women were 20–49 years old (hence of reproductive age), not pregnant, out of menstruation, and had no prior common sexually transmitted infections (STIs) including HPV, *Treponema pallidum*, and *gonococcus*. During routine gynecological inspection, female vaginal discharge was collected for *C. trachomatis* screening (Chen et al., 2020), leukorrhea routine detection, cytokine measurement, and vaginal microbiota analysis. Women who were infertile and *C. trachomatis*-positive were asked to revisit 60 days after treatment.

### Clinical Samples

All participants were asked to abstain from unprotected sex or vaginal lavage for 48 h before sampling and avoid taking antibiotics and/or antiviral drugs in the 2 weeks prior to sampling. The peeping tube was not coated with lubricant when removing secretions. Four vaginal swabs were collected by doctors or trained nurses: (1) one for *C. trachomatis* screening to be completed within 24 h; (2) one for leukorrhea routine detection to be completed within 4 h; (3) one for vaginal flora diversity analysis, stored at -80°C until use; and (4) one for cytokine measurement, stored at -20°C until use.

### Leukorrhea Routine Detection

Place and mix the vaginal swab vigorously in a tube with 1 ml sterile phosphate-buffered saline (PBS). A “wet mount” can be made with one to two drops of the vaginal discharge specimen and immediately examined under an optical microscope for observation of epithelial cells, white blood cells, clue cells, *Trichomonas vaginalis*, and fungi. Cleaning degree of leukorrhea includes grade I, II, III, and IV, ≥ grade II of which are considered abnormal leukorrhea according to the guide to Clinical Laboratory Procedures of China (Shang et al., 2015). One drop of the homogeneous mixture was also transferred to a grease-free dry slide to make a smear, and the dry smear was fixed by passing the slide quickly through a flame 3–4 times with the smear side facing up. The smear was then stained, following the instructions of the Gram Staining Kit (Baso, ZhuHai, China), to observe bacterial morphology.

### Cytokine Measurements

Each vaginal swab was placed in a tube with 1 ml PBS and centrifuged at 12,000 *g*, 30 min at 4°C. Tumor necrosis factor (TNF)-α, interleukin (IL)-6, interferon (IFN)-γ, and IL-10 in the resulting supernatant were measured using ELISA kits (Biolegend, CA, USA) following the manufacturer’s instructions. Final cytokine concentrations were calculated in pg/ml from a standard curve based on prepared dilutions of recombinant cytokines in each experiment (Chen et al., 2017).

### 16S rRNA Amplicon Sequencing

Genomic DNA was extracted by using a Genomic DNA Mini Preparation Kit (Tiangen, Beijing, China), and the resulting concentration was diluted to 1 ng/μl with sterile water. The V3–V4 hypervariable regions of 16S rRNA genes were amplified by PCR using specific primers (806R 5′-GGACTACNNGGGTATCTAAT-3′, 341F 5′-CCTAYGGGRBGCASCAG-3′) with a barcode. All reactions were conducted in a 30-µl volume comprising 0.2 µM forward and reverse primers, 15 µl PCR Master Mix, and 10 ng DNA template. The PCR conditions were initially one cycle of 1 min at 98°C; 30 cycles of 10 s at 98°C, 30 s at 50°C, and 30 s at 72°C, followed by a final extension at 72°C for 10 min.

Dominant PCR products of 400–450 bp were selected for further experiments and were mixed in equidensity ratios, purifying with the GeneJET Gel Extraction Kit (Thermo Scientific, USA). Following the manufacturer’s recommendations, sequencing libraries were conducted using Library Prep Kit for Illumina, and index codes were added. Library quality was assessed on the Agilent Bioanalyzer 2100 system and Qubit@ 2.0 Fluorometer (Thermo Scientific, USA). The library was ultimately sequenced on an Illumina HiSeq platform generating 250-bp paired-end reads.

### Data Analysis

The operational taxonomic units (OTUs) clustering and species classification were analyzed at 97% identity level based on effective data discarding low-quality reads. Each OTU was annotated on the basis of OTU clustering to obtain the species-based abundance distribution and corresponding species information. The relative abundance, alpha-diversity calculation, Venn plot, and petal plot analysis of OTUs were carried out to obtain the information of species richness and evenness within the sample, as well as the information of common and special OTUs among different groups/samples. As well, multisequence alignment of OTUs can be carried out and phylogenetic trees can be constructed. Through dimension-reduction analysis such as principal coordinate analysis (PCoA), principal component analysis (PCA), nonmetric multidimensional scaling (NMDS), and sample cluster tree, the differences between community structure among distinct samples or groups can be explored. To further determine the differences in community structure among grouped samples, statistical analysis methods such as Student’s *t*-test, Simper, Metastat, Linear discriminant analysis Effect Size (LEfSe), analysis fo similarities (Anosim), and multi-response permutation procedure (MRPP) were used to test the significance of differences in species composition and community structure among samples.

### qRT-PCR

Bacterium-specific qRT-PCR assays were conducted by amplifying species-specific regions of the 16S rRNA gene. Primers specific to 17 dominant microorganisms characterized by the 16S rRNA amplicon were designed or obtained from the literature (Blackwood et al., 2005; Yang et al., 2015). Each subject was amplified with all primer pairs and was subjected to a human β-globin PCR to ensure the quality of amplifiable DNA and to monitor for PCR inhibitors. Each 20-μl qRT-PCR mixture contained 10 μl 2× Taq Plus MasterMix (TaKaRa, Dalian, China), 2 μl template DNA, and 0.2 μM of each primer (**Table S1**). The PCR reaction conditions were as follows: one cycle of 5 min at 95°C, 40 cycles of 30 s at 95°C, 35 s at 60°C–65°C (depend on the melting temperature of the primers), and 45 s at 72°C, then a final extension at 72°C for 10 min. Melting curve analysis was then conducted at 95°C for 15 s, 60°C for 50 s, and 95°C for 15 s before the reaction was terminated.

Serial dilutions of 1, 1:4, 1:16, 1:64, and 1:256 vaginal bacterial total DNA were used to determine primer amplification efficiencies. Quantitative analysis of vaginal bacterial communities was performed using the following formula:

$$X=\frac{{\left(Eff\cdot Univ\right)}^{Ct\;univ}}{{\left(Eff\cdot Spec\right)}^{Ct\;spec}}\times 100$$

where “Ct spec” and “Ct univ” represent Cycle threshold, “Eff. Spec” represents the efficiency of various genus-specific primers, and “Eff. Univ” represents the efficiency of bacterial universal primers. “X” represents the percentage of bacterial species-specific gene copy number existing in a sample.

### Statistical Analysis

Statistical analyses were conducted using GraphPad Prism 8.0 and SPSS 22.0. Differences of age in the study population were tested using a Student’s *t*-test, and leukorrhea characteristics were analyzed using the chi-square, Fisher’s exact tests. One-way analysis of variance in combination with Tukey’s *post-hoc* tests was used to determine the differences in univariate data between the samples including cytokine measurements, DNA yield, Bray–Curtis dissimilarity, richness, and qRT-PCR results. Statistical significance of alpha-diversity measures was calculated using Qiime software, and beta-diversity parameters were determined using the R Studio package “phyloseq.”

## Results

### Characteristics of Study Subjects

Thirty samples from 26 women of reproductive age enrolled in the study were obtained. These were nine women who were CT-N and eight women who were CT-P from ART plus nine women classed as CT-C from PEC. Two CT-P participants discovered to be pregnant during the sampling visit were excluded from the study.

Five samples (two CT-C, one CT-N, and two CT-P samples) were excluded from 16S rRNA amplicon-based sequencing because of their failure to pass the sequencing and quality control. Consequently, 25 cervical samples comprising seven CT-C, eight CT-N, six CT-P, and four CT-PT can be applicable for downstream analysis. No significant differences were observed in the age distribution between the different groups of women (**Table 1**).

**Table 1:** Leukorrhea routine detection and characteristics of the study population.

Classification	CT-C (n = 7)	CT-N (n = 8)	CT-P (n = 6)	CT-PT (n = 4)	p
Age, years	33.6 ± 3.7	33.9 ± 4.2	34.1 ± 4.8	33.8 ± 4.6	>0.05
Cleaning degree					>0.05
I	3 (42.8%)	4 (50.0%)	0 (0%)	1 (25.0%)	
II	2 (28.6%)	3 (37.5%)	2 (33.3%)	2 (50.0%)	
III	2 (28.6%)	1 (12.5%)	2 (33.3%)	1 (25.0%)	
IV	0 (0%)	0 (0%)	2 (33.3%)	0 (0%)	
Leukocyte					>0.05
I	5 (71.4%)	6 (75.0%)	2 (33.3%)	3 (75.0%)	
II	2 (28.6%)	1 (12.5%)	2 (33.3%)	1 (25.0%)	
III	0 (0%)	1 (12.5%)	2 (33.3%)	0 (0%)	

CT-C, healthy women with C. trachomatis-negative from the PEC; CT-N, infertile women with C. trachomatis-negative from the ART; CT-P, infertile women with C. trachomatis-positive from the ART; CT-PT, CT-P women post-treatment with azithromycin; ART, assisted reproductive technology center; PEC, physical examination center.

### Leukorrhea and Cytokine Production in Vaginal Discharge

Leukorrhea abnormal features were found in 66.6% (4/6) of the samples in the CT-P group but only in 28.6% (2/7) for CT-C, 12.5% (1/8) for CT-N, and 25.0% (1/4) for CT-PT groups. However, there were no significant differences between the groups (p > 0.05). Similar results were found for lekcorrhea leukocytes (**Table 1**). Fewer vaginal bacterial species were observed in the CT-P group samples compared with the CT-C and CT-N groups, but this was restored to some extent following treatment (**Figure S1**).

**Figure 1:**
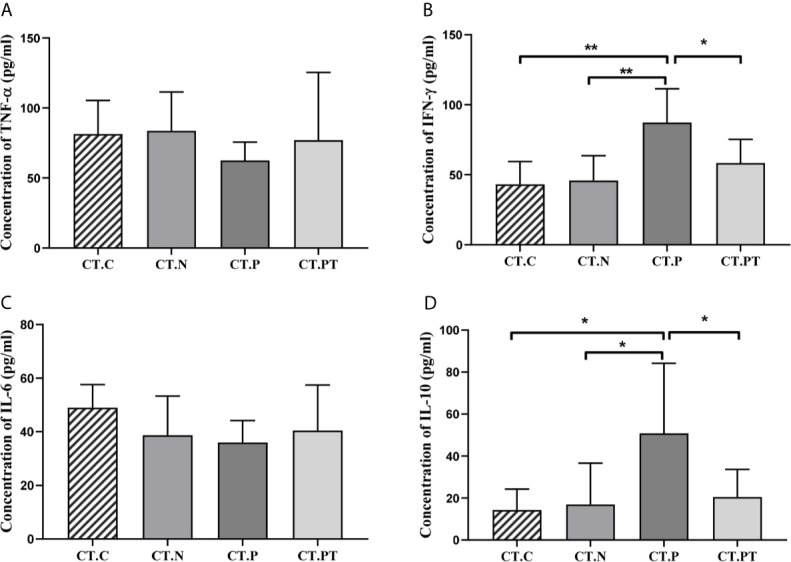
Cytokine production in vaginal secretions in each group. **(A)** Tumor necrosis factor (TNF)-α, **(B)** interferon (IFN)-γ, **(C)** interleukin (IL)-6, and **(D)** IL-10 levels were detected in vaginal discharge samples using ELISA kits. Each bar represents the mean ± SD of the cytokine levels (pg/ml). *p < 0.05 and **p < 0.01. CT-C, women from the physical examination center (PEC) who were healthy and *C. trachomatis*-negative; CT-N, women who were infertile and *C. trachomatis*-negative from the assisted reproductive technology center (ART); CT-P, women who were infertile and *C. trachomatis*-positive from the ART; CT-PT, CT-P women post-treatment with azithromycin.

Vaginal inflammation induced by *C. trachomatis* was further investigated by measuring the production of IFN-γ, TNF-α, IL-6, and IL-10. There were significantly higher levels of IFN-γ and IL-10 in the CT-P group than in the CT-C, CT-N, and CT-PT groups (p < 0.05), while there were no significant differences in TNF-α and IL-6 levels (p > 0.05; **Figure 1**). This indicated that *C. trachomatis* genital tract infection might induce a local inflammatory response.

### Overview of the Vaginal Microbiota Sequencing Results

Illumina Nova sequencing was applied to determine the vaginal microbiota diversity. A total of 1,949,887 raw tags were obtained. After preprocessing, there was a final average of 65,600 high-quality sequences per sample for downward analysis, which clustered into 4,385 OTUs at the 97% similarity level for further species annotation. The number of shared OTUs among the samples was 221, which accounted for 5.4% of the 4,384 total OTUs (**Figure S2**), suggesting that there were large differences in the populations during *C. trachomatis* infection. In addition, the Good’s coverage index value of each sample exceeded 98% (**Table 2**), suggesting the sequencing depth covered over 98% of the bacterial phylotypes and precisely reflected the microorganisms contained in each sample. The plot species accumulation curve became an asymptote after a sharp rise (**Figure S3**). This slow rise indicated that the species in this environment would not increase significantly with an increase in sample size and suggested that additional data volume would only produce a small number of new species. This further verified that the sampling and sequencing data were reasonable and sufficient.

**Table 2:** Alpha-diversity index for each group.

Group	Observed species	Shannon	Simpson	Chao1	ACE	Good’s coverage
CT-C	427.86 ± 182.55	2.34 ± 1.44	0.53 ± 0.29	777.73 ± 327.01	892.58 ± 377.19	0.99 ± 0.00
CT-N	384.88 ± 374.46	1.82 ± 1.14	0.41 ± 0.27	689.01 ± 550.35	737.67 ± 532.83	0.99 ± 0.01
CT-P	258.33 ± 210.96*	1.32 ± 1.29*	0.32 ± 0.36	468.74 ± 469.55	559.80 ± 677.34	0.99 ± 0.01
CT-PT	243.25 ± 164.68*	1.59 ± 1.02	0.33 ± 0.33	491.67 ± 353.47	575.41 ± 410.79	0.99 ± 0.00

CT-C, women from the PEC who were healthy and C. trachomatis-negative; CT-N, women who were infertile and C. trachomatis-negative from the ART; CT-P, women who were infertile and C. trachomatis-positive from the ART; CT-PT, CT-P women post-treatment with azithromycin; ART, assisted reproductive technology center; PEC, physical examination center; ACE, abundance-based coverage estimators. *p < 0.05 compared with CT-N.

### Alpha- and Beta-Diversity Analyses

To evaluate the richness and diversity of vaginal microbial communities in samples, alpha-diversity analyses were conducted. Alpha-diversity metrics, including Shannon and observed species indices, indicated significant differences in biodiversity between the different groups (p < 0.01 and p < 0.05). A significantly lower Shannon index was observed in CT-P samples compared with CT-C (p < 0.01) and CT-N (p < 0.05) samples (**Table 2**), indicating that genital infection with *C. trachomatis* may be associated with a decreased diversity of the vaginal microbiota in women who were infertile. However, there were no statistical differences in Simpson, Chao1, and abundance-based coverage estimators (ACE) (**Table 2**).

**Figure 2:**
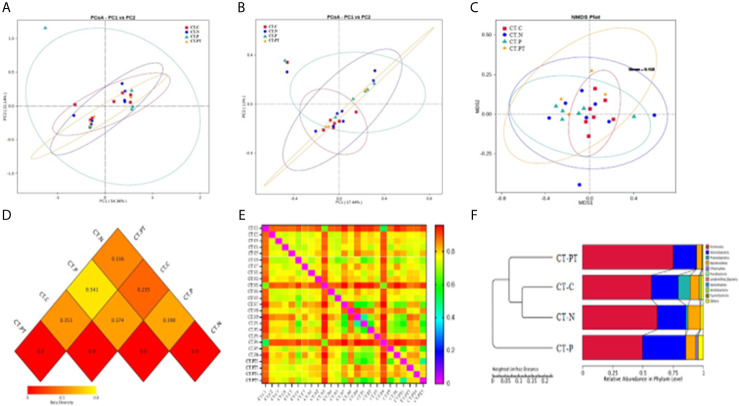
Comparative analysis of beta diversity. Principal coordinate analysis (PCoA) based on weighted UniFrac **(A)** and unweighted Unifrac **(B)** distance. **(C)** Nonmetric multidimensional scaling analysis of each sample. Heatmap of beta-diversity index for each group **(D)** and samples **(E)**; the legend below the heatmap represents each participant. **(F)** Unweighted pair group method with arithmetic mean (UPGMA) clustering tree of each group. CT-C, women from the physical examination center (PEC) who were healthy and *C*. *trachomatis*-negative; CT-N, women who were infertile and *C*. *trachomatis*-negative from the assisted reproductive technology center (ART); CT-P, women who were infertile and *C. trachomatis*-positive from the ART; CT-PT, CT-P women post-treatment with azithromycin.

Beta diversity was employed to understand the divergence in community composition between samples. PCoA based on Bray–Curtis dissimilarities showed no significant segregation of the groups on either weighted (**Figure 2A**) or unweighted Unifrac distance (**Figure 2B**). However, the CT-P vaginal samples were clustered and separated from the other samples when subjected to NMDS analysis based on unweighted Unifrac distance (**Figure 2C**). Moreover, a heatmap of beta-diversity index (**Figures 2D, E**) revealed that there was a statistically significant species diversity of CT-P compared with the other groups (p < 0.01). The unweighted pair group method with arithmetic mean (UPGMA) clustering tree, using Euclidean distance matrices with Ward linkage (**Figure 2F**), confirmed that vaginal samples in the CT-P group exhibited a tendency toward clustering and relatively diverged from the samples from the CT-C, CT-N, and CT-PT groups. This suggested that women in the CT-P group possessed a unique microbial composition that differed from that of healthy women, and that alterations in relative taxa abundance occurred after treatment for *C. trachomatis* infection.

### Taxonomic Composition of Vaginal Microbiota

**Figure 3:**
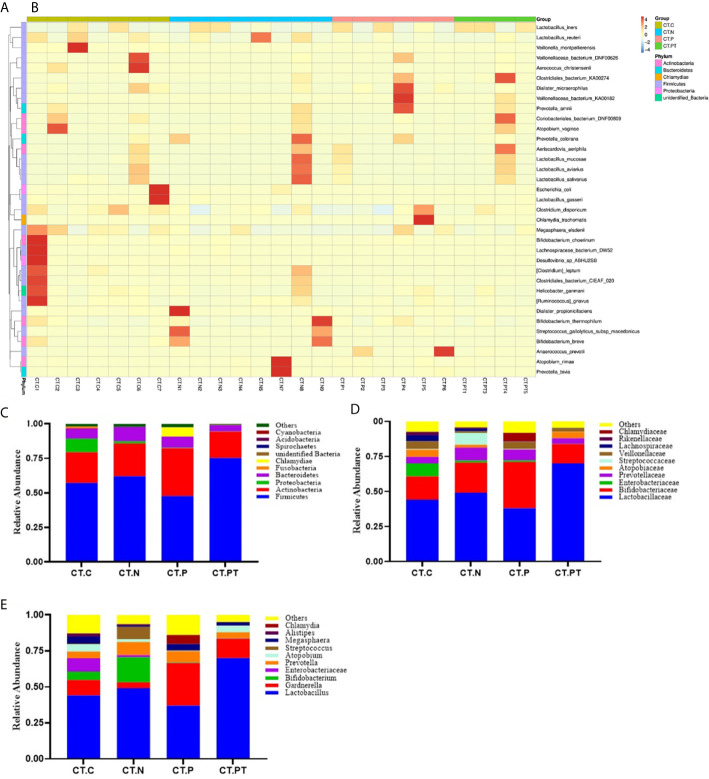
Taxonomic composition of the vaginal microbiota. **(A)** Complete linkage clustering of sample relationships generated from Bray–Curtis dissimilarity matrix. **(B)** Heatmap of the log10-transformed proportions of the top 35 bacterial taxa, in terms of relative abundance, found in the vaginal microbiota. Stacked bar charts of top 10 taxa in terms of relative abundances at **(C)** phylum, **(D)** family, and **(E)** genus level for each group. Remaining taxa are grouped in the “Other” category. CT-C, women from the physical examination center (PEC) who were healthy and *C. trachomatis*-negative; CT-N, women who were infertile and *C*. *trachomatis*-negative from the assisted reproductive technology center (ART); CT-P, women who were infertile and *C*. *trachomatis*-positive from the ART; CT-PT, CT-P women post-treatment with azithromycin.

Bacterial taxa identified in the 25 studied vaginal samples comprised a total of 21 phyla, 30 classes, 66 orders, 115 families, and 203 genera. To further explore the taxonomic composition of vaginal microbiota in each group, the phylotypes were clustered according to their correlation profiles and the vaginal bacterial communities were grouped according to community composition. The heatmap in **Figures 3A, B** representing log10-transformed relative abundances of microbial taxa highlights the variation of microbial composition among the individuals in the study and the diversity in all vaginal bacterial communities. Of the 25 samples, 10 had *L. iners*-dominated microbiota and the rest had a community dominated by *Lactobacillus reuteri*, *Bifidobacterium breve*, *Lactobacillus gasseri*, *Atopobium vaginae*, or Clostridiales bacterium.

At the phylum level, the vaginal microbiota of all women was mainly dominated by Firmicutes, with abundance ranging from 75.2% in CT-PT to 48.8% in CT-P, followed by Actinobacteria, Proteobacteria, Bacteroidetes, Fusobacteria, Chlamydiae, Spirochaetes, and Acidobacteria, accounting for an average of 0.1%–25.1% of the total relative abundance (**Figure 3C**). This was congruent with previously reported vaginal microbiota types (Filardo et al., 2017). Moreover, the relative abundance of the phylum Firmicutes was reduced in women who were *C. trachomatis*-positive when compared with participants in the CT-C (57.1%) and CT-N (61.9%) groups. Conversely, the phylum Acidobacteria was significantly enriched in the CT-P group (34.4%) compared with the CT-C (22.2%), CT-N (23.7%), and CT-PT (19.1%) groups. No statistically significant differences were observed in relation to phylum distribution between the CT-C group and the CT-N group.

At the family taxonomic level, Lactobacillaceae, as well as Enterobacteriaceae, Prevotellaceae, Atopobiaceae, Streptococcaceae, Veillonellaceae, and Lachnospiraceae, were less abundant in CT-P samples than in CT-N samples. In contrast, Bifidobacteriaceae was more abundant in CT-P samples compared with CT-N samples (median relative abundance 33.1% and 21.4%, respectively; **Figure 3D**).

Analysis of the vaginal microbiota composition at the lower taxonomic level of the genus revealed that eight out of the top 10 taxa in terms of relative abundance were significantly different between CT-N and CT-P samples (**Figure 3E**). These included *Lactobacillus*, *Bifidobacterium*, *Enterobacter*, *Atopobium*, *Streptococcus*, and *Alistipes*, which were all more abundant in the CT-N samples, and *Chlamydia*, *Gardnerella*, and *Megasphaera*, which were more abundant in CT-P samples, suggesting that these bacteria, especially *Lactobacillus*, might be predictive of *C. trachomatis* infection. These results were in agreement with findings that growing strict and facultative anaerobes and depleted of *Lactobacillus* in vaginal microbiota could increase the risk of *C. trachomatis* infection (Balle et al., 2018). Notably, vaginal samples from the CT-PT group showed a robust increase in *Lactobacillus* (70.0%) compared with CT-P samples (37.1%), and this was even higher than that of CT-N samples (49.2%). Furthermore, other microorganisms including *Bifidobacterium*, *Enterobacter*, *Atopobium*, and *Streptococcus* were also restored with varying degrees in the CT-PT samples. Moreover, no genome sequences from *Chlamydia* were detected in the CT-PT samples, indicating the great effectiveness of treatment of *C. trachomatis* infection with azithromycin.

### Alterations of Core Microbiomes in Women With Infertility and *C. trachomatis* Infection

**Figure 4:**
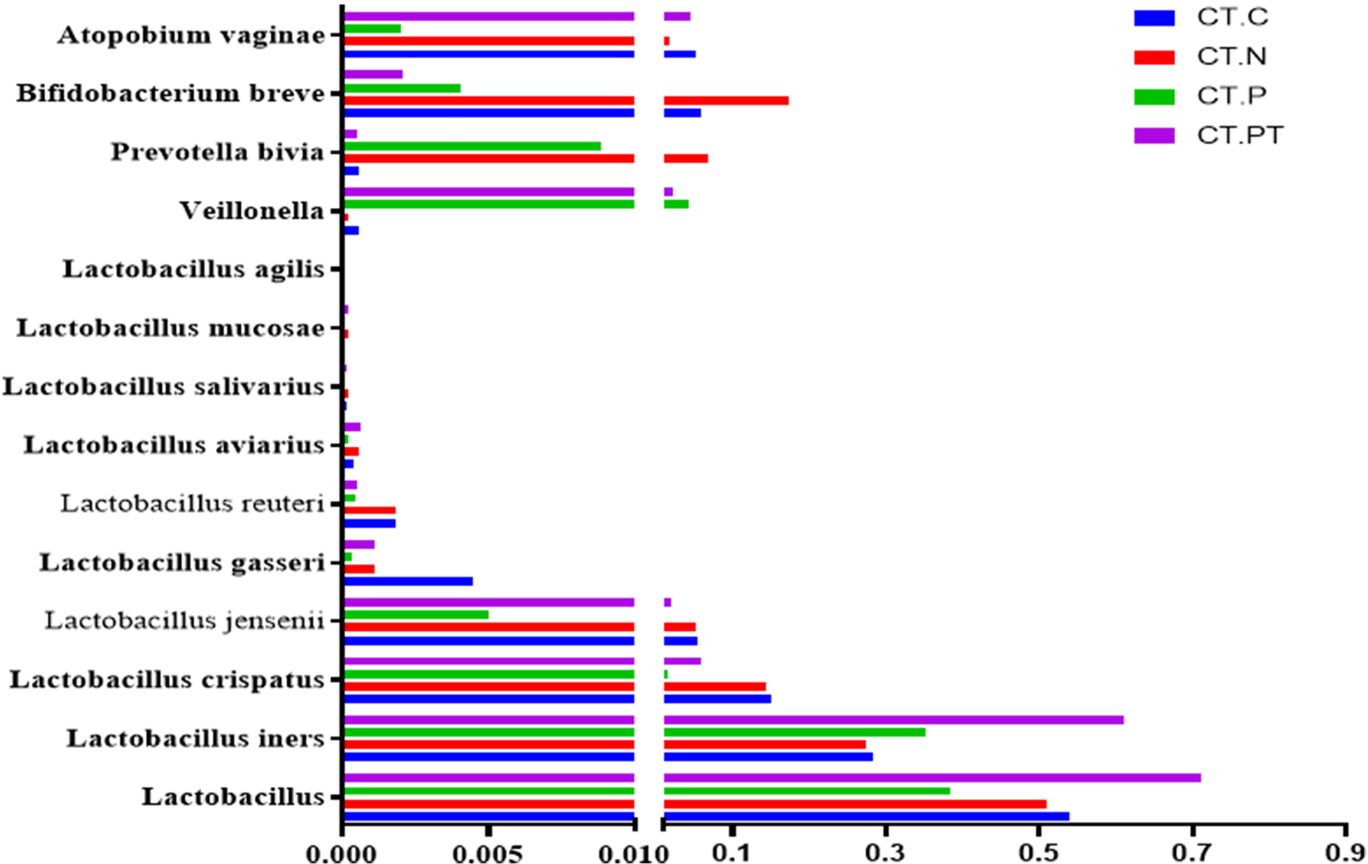
Detection of dominant vaginal flora by specific qRT-PCR. CT-C, women from the physical examination center (PEC) who were healthy and *C. trachomatis*-negative; CT-N, women who were infertile and *C. trachomatis*-negative from the assisted reproductive technology center (ART); CT-P, women who were infertile and *C. trachomatis*-positive from the ART; CT-PT, CT-P women post-treatment with azithromycin.

To verify the predictive core microbiomes of *C. trachomatis* infection beyond the vagina, a bacterium-specific qRT-PCR approach capable of normalizing the variation in the absolute DNA yields of each sample was employed. As shown in **Figure 4**, the genus *Lactobacillus* dominated the vaginal microbiota of all women, with abundances ranging from 75.2% in CT-PT samples to 48.8% in CT-P samples. The abundances of most *Lactobacillus*, including *L. crispatus*, *L. gasseri*, *L. jensenii*, *L. reuteri*, and *L. aviaries* and *B. breve*, *Prevotella bivia*, and *A. vaginae* in CT-P samples showed a decrease with respect to CT-C, CT-N, or CT-PT samples, whereas the proportions of *L. iners* and *Veillonellaceae bacterium* KA00182 were significantly increased in CT-P samples compared with CT-C and CT-N samples. This was in agreement with corresponding high-throughput 16S rRNA gene amplicon sequencing data (**Figure 3**).

## Discussion

*C. trachomatis* is recognized as a leading cause of infertility in women (Nsonwu-Anyanwu et al., 2015). Recent *in vivo* studies demonstrated the link between vaginal microbiota dysbiosis and *C. trachomatis* infection (van Houdt et al., 2018). The present study firstly investigated the associations between vaginal microbiota and female infertility with *C. trachomatis* infection. Participants enrolled in this study included women with tubal infertility and proven *C. trachomatis* infection pre- and post-antibiotic treatment, women with tubal infertility who were *C. trachomatis*-negative, and women who were deemed healthy and *C. trachomatis*-negative. The vaginal microbiota composition in each group of women was analyzed using next-generation sequencing of 16S rRNA gene amplicons, and the diversity and richness of vaginal microbiota from women with tubal infertility and *C. trachomatis* infection were compared with those without such infection. Data from this study highlighted notable alterations in the vaginal microbiota in women with tubal infertility and *C. trachomatis* infection. In particular, the vaginal microbiota of these women tended to show a decrease in *Lactobacillus* species dominated by *L. iners* rather than *L. crispatus* compared with control samples. However, samples from women with tubal infertility and *C. trachomatis* infection treated with azithromycin showed a significant increase in *Lactobacillus* species dominated by *L. iners*.

The current study did not reveal any significant differences in phylum, class, and OTU levels between women with tubal infertility who were *C. trachomatis*-negative and healthy controls, suggesting an equivalent baseline of overall bacterial community compositions among these women. This may explain the *L. iners*-dominated vaginal microbiota in women with infertility and *C. trachomatis* infection, as well as in healthy women of reproductive age with *C. trachomatis* infection (Edwards et al., 2019). Similarly, van Houdt et al. (2018) emphasized that *L. iners*-dominated vaginal community could increase the risk for acquiring *C. trachomatis* genital infection among Dutch women. As anticipated, most vaginal microorganisms in CT-P women were also recovered to varying degrees when treated with azithromycin (Tamarelle et al., 2020). Surprisingly, *L. iners* accounted for more than 70% of the total vaginal bacteria among them, which was supposed to be at the same level as that in healthy controls (about 45%). This might be attributed to the high level of azithromycin resistance in *L. iners* (Edwards et al., 2019; Tamarelle et al., 2020). These findings support the postulation that *L. crispatus* is associated with stronger protection than *L. iners* against *C. trachomatis* infection (Caselli et al., 2020).

The vagina is an elastic, yet muscular, canal that harbors a large number of microorganisms including *Lactobacillus*, *Streptococcus*, *Staphylococcus*, *Escherichia coli*, and several anaerobes, which compose the vaginal microecosystem and maintain its self-cleaning property (Gupta et al., 2019). However, the vaginal microbiota varies greatly among individuals due to host intrinsic factors such as age, diet, ethnicity, menstrual cycle, and external factors such as geographic location and genital diseases (Mendling, 2016). This variation was also further confirmed by the within-sample diversity of vaginal microbiota found in the current study and could explain the outliers following dimension-reduction analysis.

To acquire a deeper insight into the variability in overall bacterial community compositions among CT-C, CT-N, CT-P, and CT-PT subjects, alpha diversity was applied to measure the average species diversity within a sample community and beta diversity was applied to test the divergence in community composition. A significantly different microbial diversity was found between the CT-N group and the CT-P group, as evidenced by robust decreases in Shannon and observed species indices among *C. trachomatis*-infected women, as well as a decrease in other alpha-diversity metrics (Simpson, Chao1, and ACE), although these were not significant. These observations of alpha diversity were coupled with greater beta diversity compared with healthy controls and were also consistent with the previously reported similar alpha diversity of endocervical microbiota between women who were *C. trachomatis*-positive and those who were uninfected (Balle et al., 2018).

In the current study, each group could be characterized by a unique fingerprint as evidenced by the variation presented in the relative abundance of each taxon, although the taxa composition is basically the same, in accordance with a pilot study indicating comparable taxa composition between women who were *C. trachomatis*-positive and healthy subjects (Filardo et al., 2017). For example, Lactobacilli, thought to be instrumental in host defense of the vagina and ectocervix owing to their ability to produce lactic acid, were frequently found in all female vaginal microbiota in the current study and ranged from 70.0% to 45% in individuals (Edwards et al., 2019). The sequencing technology applied to this study could not exactly reflect all the “species” and “strains” of the microbial community (Yang et al., 2015); thus, a species-specific qRT-PCR was employed to verify the abundances of dominant vaginal bacteria Lactobacilli. The data demonstrated a reasonable concordance with amplicon sequencing findings that *L. iners* and *V. bacterium* were increased, and *L. crispatus*, *L. jensenii*, *L. gasseri*, *L. reuteri*, *L. aviaries*, *L. salivarius*, *L. mucosae*, *L. agilis*, *P. bivia*, *B. breve*, and *A. vaginae* decreased in the vaginal microbiota of women with tubal infertility who were *C. trachomatis*-positive. These data further confirmed the evidence that *L. iners*-dominated vaginal microbiota strongly increased the risk for genital *C. trachomatis* infection (van Houdt et al., 2018). This may be due to the fact that *L. iners* is incapable of downregulating histone deacetylase 4 and does not sufficiently reduce cell proliferation to protect against *C. trachomatis* infection (Petrova et al., 2017; Edwards et al., 2019). Conversely, other species of the genus *Lactobacillus* such as *L. jensenii*, *L. crispatus*, and *L. gasseri* are capable of producing D-lactic acid, bacteriocins, and other antimicrobial compounds to protect against sexually transmitted pathogens, including *C. trachomatis*, *Neisseria gonorrhoeae*, and HPV (Brotman et al., 2014; Placzkiewicz et al., 2020). In particular, *L. crispatus* was reported to suppress the adhesion and infectivity of *C. trachomatis* in human epithelial cells (Nardini et al., 2016).

*C. trachomatis* is an intracellular pathogen that generally triggers a strong host T-helper 1 (Th1) cell and IFN-γ response by the release of chemokines upon infection, and in turn, this could magnify the inflammatory response by recruiting *Chlamydia*-specific immune cells (Elwell et al., 2016; Vicetti Miguel et al., 2016). In addition, *C. trachomatis* mediated production of IL-10 both *in vitro* and *in vivo* (Ohman et al., 2006). Therefore, it is not surprising that in the current study, women with tubal infertility who were *C. trachomatis*-positive had significantly higher vaginal levels of IFN-γ and IL-10 compared with those in healthy control subjects.

There were numerous strengths of the current study. Firstly, the strict inclusion criteria for population selection greatly diminished the impact of the confounding bias, allowing a clear judgment to be made. Secondly, both next-generation sequencing and species-specific qRT-PCR technology were applied to guarantee the accuracy of the relative abundance of taxa in vaginal microbiota. A third strength is that women with tubal infertility who were *C. trachomatis*-positive were enrolled pre- and post-antibiotic treatment, which facilitated a better understanding of the relationship between vaginal microbiota and *C. trachomatis* infection. However, the vaginal microbiota composition prior to *C. trachomatis* infection in these participants is not known and this is a limitation of the study. The study is also limited by the low number of participants, precluding exploration of the unique taxa directly associated with *C. trachomatis* infection.

In summary, this study provides the first demonstration that women with tubal infertility and *C. trachomatis* infection are prone to have an *L. iners*- rather than *L. crispatus*-dominated vaginal microbiota and have a decrease in *Lactobacillus*, *Bifidobacterium*, *Enterobacter*, *Atopobium*, and *Streptococcus*, which could be restored with varying degrees by azithromycin treatment. Findings from the study contribute valuable information for epidemiological and fundamental research on *C. trachomatis* and further illuminate the potential of probiotics treatment for *C. trachomatis* infection.

## Data Availability Statement

The original contributions presented in the study are included in the article/**Supplementary Material**. The 16S rRNA amplicon sequencing data presented in this study are available in the GenBank sequence database, accession number PRJNA725638. Further inquiries can be directed to the corresponding author.

## Ethics Statement

This study was approved by the Ethics Committee of Chenzhou No. 1 People’s Hospital (no. CZ/1128). This study was performed in accordance with the Declaration of Helsinki.

## Author Contributions

Conceptualization: HC, LW, LZ and ZL. Methodology: LW, LL, SM and LZ. Software: HC and LW. investigation: HC, LZ and SM. Data curation: LW, LL, SM, YW, WL, and MS. Writing—original draft preparation: HC and LW. Writing—review and editing: ZL. Funding acquisition: HC and ZL. All authors contributed to the article and approved the submitted version.

## Funding

This work was funded by the NSFC (81802022 and 81772210), Hunan Health Commission Project (20200282), and Chenzhou No. 1 People’s Hospital Project (yfzx201908 and N2019-004).

## Conflict of Interest

The authors declare that the research was conducted in the absence of any commercial or financial relationships that could be construed as a potential conflict of interest.

## Publisher’s Note

All claims expressed in this article are solely those of the authors and do not necessarily represent those of their affiliated organizations, or those of the publisher, the editors and the reviewers. Any product that may be evaluated in this article, or claim that may be made by its manufacturer, is not guaranteed or endorsed by the publisher.
