# Supplementary material for: Alterations of Vaginal Microbiota in Women With Infertility and Chlamydia trachomatis Infection
Source: Front Cell Infect Microbiol. 2021 Aug 3;11:698840. doi: 10.3389/fcimb.2021.698840 (PMC8370387; doi:10.3389/fcimb.2021.698840)
Supplement: Supplementary file 1 [file Table_1.docx]

**Supplementary Materials**

**Table S1 Specific primers and universal primers for vaginal bacteria**

| Genus | Primers | Primer sequence | Annealing temperature |
| --- | --- | --- | --- |
| Universal primer | 926 F | AAACTCAAAKGAATTGACGG | 65℃ |
|  | 1062 R | CTCACRRCACGAGCTGAC |  |
| *Lactobacillus* | Bact-0011 | TGGAAACAGRTGCTAATACCG | 60℃ |
|  | Lab-0677 | GTCCATTGTGGAAGATTCCC |  |
| *L.iners* | Liners F | CTCTGCCTTGAAGATCGGAGTGC | 65℃ |
|  | Liners R | ACAGTTGATAGGCATCATCTG |  |
| *L.crispatus* | Lcris F | AGCGAGCGGAACTAACAGATTTAC | 65℃ |
|  | Lcris R | AGCTGATCATGCGATCTGCTT |  |
| *L.jensenii* | Ljens F | AAGTCGAGCGAGCTTGCCTATAGA | 60℃ |
|  | Ljens R | CTTCTTTCATGCGAAAGTAGC |  |
| *L.gasseri,* | Lgass F | TGGAAACAGRTGCTAATACCG | 60℃ |
|  | LgassR | CAGTTACTACCTCTATCTTTCTTCAC  TAC |  |
| *L.reuteri* | Lreu F | ACCGAGAACACCGCGTTATTT | 60℃ |
|  | Lreu R | CATAACTTAACCTAAACAATCAAAG  ATTGTC |  |
| *L.mucosae* | Lmuc F | GGCCGTTACCCTACCAACAA | 60℃ |
|  | Lmuc R | CTTGCACGGACTTGACGTTG |  |
| *L.salivarius* | Lsali F | CGAAACTTTCTTACACCGAATGC | 62℃ |
|  | Lsali R | GTCCATTGTGGAAGATTCCC |  |
| *L. avium* | Ava F | TATCGCTTTTGGATGGAC | 60℃ |
|  | Ava R | TGGTTAGATACCGTCAGT |  |
| *Gardnerella vaginalis* | GV F2 | GGGCGGGCTAGAGTGCA | 60℃ |
|  | GV R2 | GAACCCGTGGAATGGGCC |  |
| *Atopobium*  *vaginae* | Ato-v F | TAGGTCAGGAGTTAAATCTG | 62℃ |
|  | Ato-v R | TCATGGCCCAGAAGACCGCC |  |
| *L.agilis* | Lagilis F | CGTCAAGTCATCATGCCCCT | 62℃ |
|  | Lagilis R | CACCTTAGACGGCTAGCTCC |  |
| *Prevotella bivia* | P-bivia F2 | TGTGAGGAAGGTGGGGATGA | 62℃ |
|  | P-bivia R2 | GGTCGATCCTTACGGTCACG |  |
| *Vellonella* | V 00182 F | ACCGTAAGAGAAAGCCACGG | 60℃ |
|  | V 00183 R | CTTCCAGTTTCGGTCCCCTC |  |
| *Bifidobacterium breve* | B-bre F | ACTGAGATACGGCCCAGACT | 60℃ |
|  | B-bre R | AACACAAAGTGCCTTGCTCC |  |
| β-globin | B-GH20 F | GAAGAGCCAAGGACAGGTAC | 60℃ |
|  | B-PC04 R | CAACTTCATCCACGTTCACC |  |

**
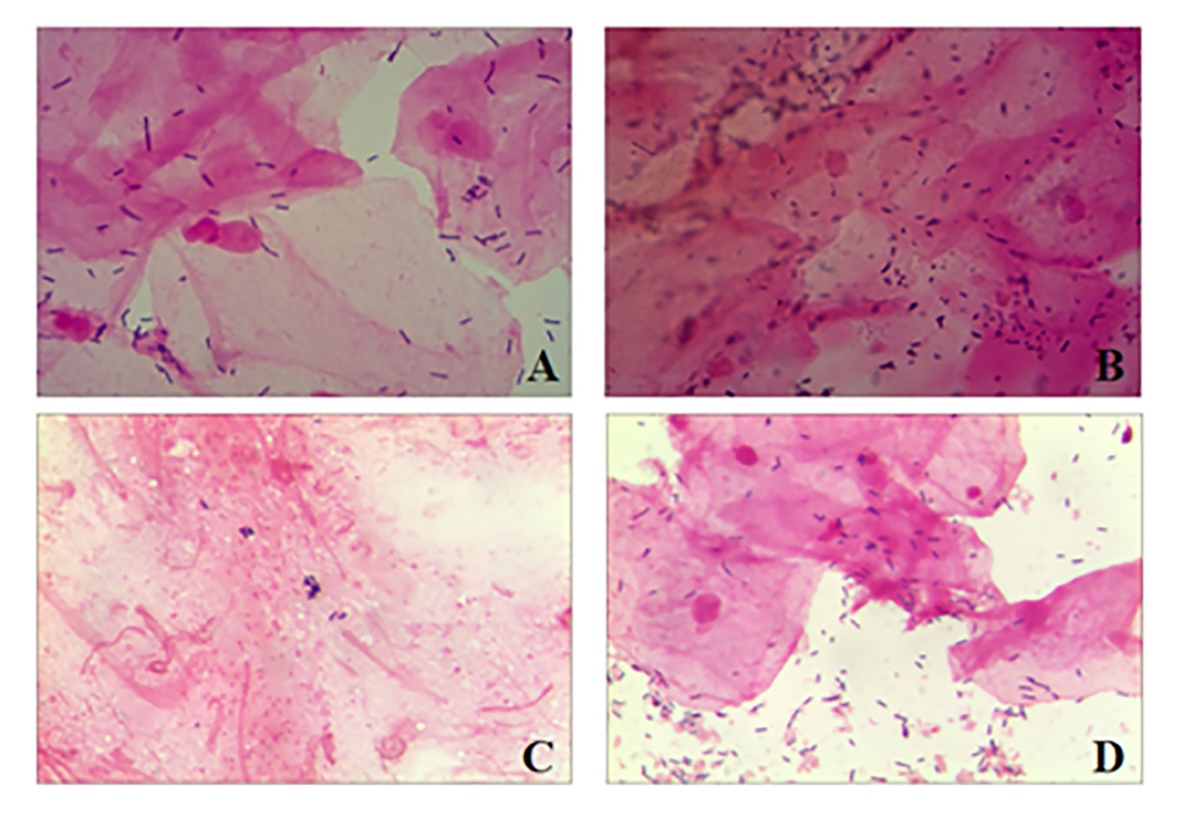
**

**Figure S1 Gram staining of vaginal secretions**

Figure S1: A: CT-C: Healthy women with C. trachomatis-negative from the physical examination center; B: CT-N: infertile women with C. trachomatis-negative from the assisted reproductive technology center; C: CT-P: infertile women with C. trachomatis-positive from the assisted reproductive technology center; D: CT-PT: CT-P women post-treatment with azithromycin.


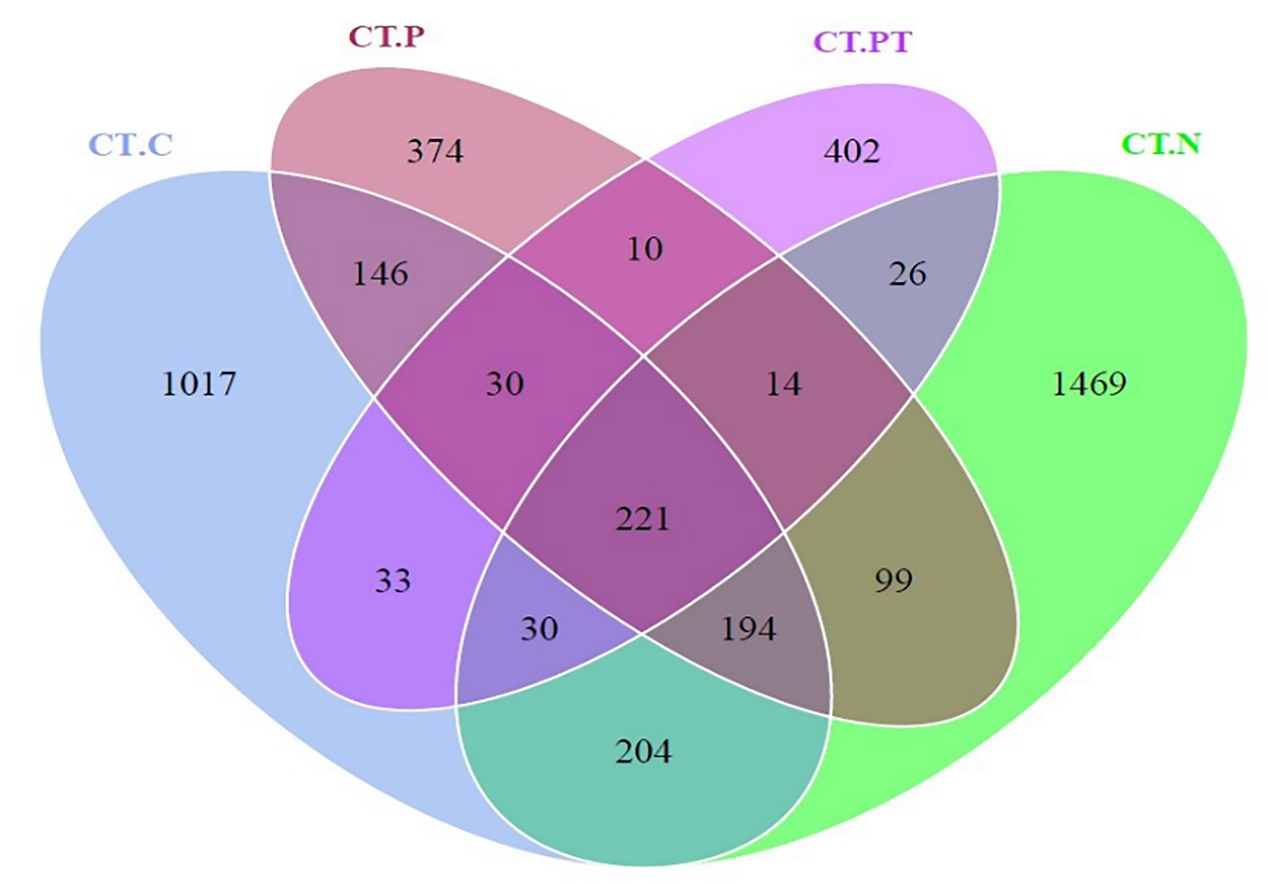


**Figure S2 Venn diagram based on OTUs among groups at 3% non-similarity level**

Figure S2: CT-C: Healthy women with C. trachomatis-negative from the physical examination center; CT-N: infertile women with C. trachomatis-negative from the assisted reproductive technology center; CT-P: infertile women with C. trachomatis-positive from the assisted reproductive technology center; CT-PT: CT-P women post-treatment with azithromycin.


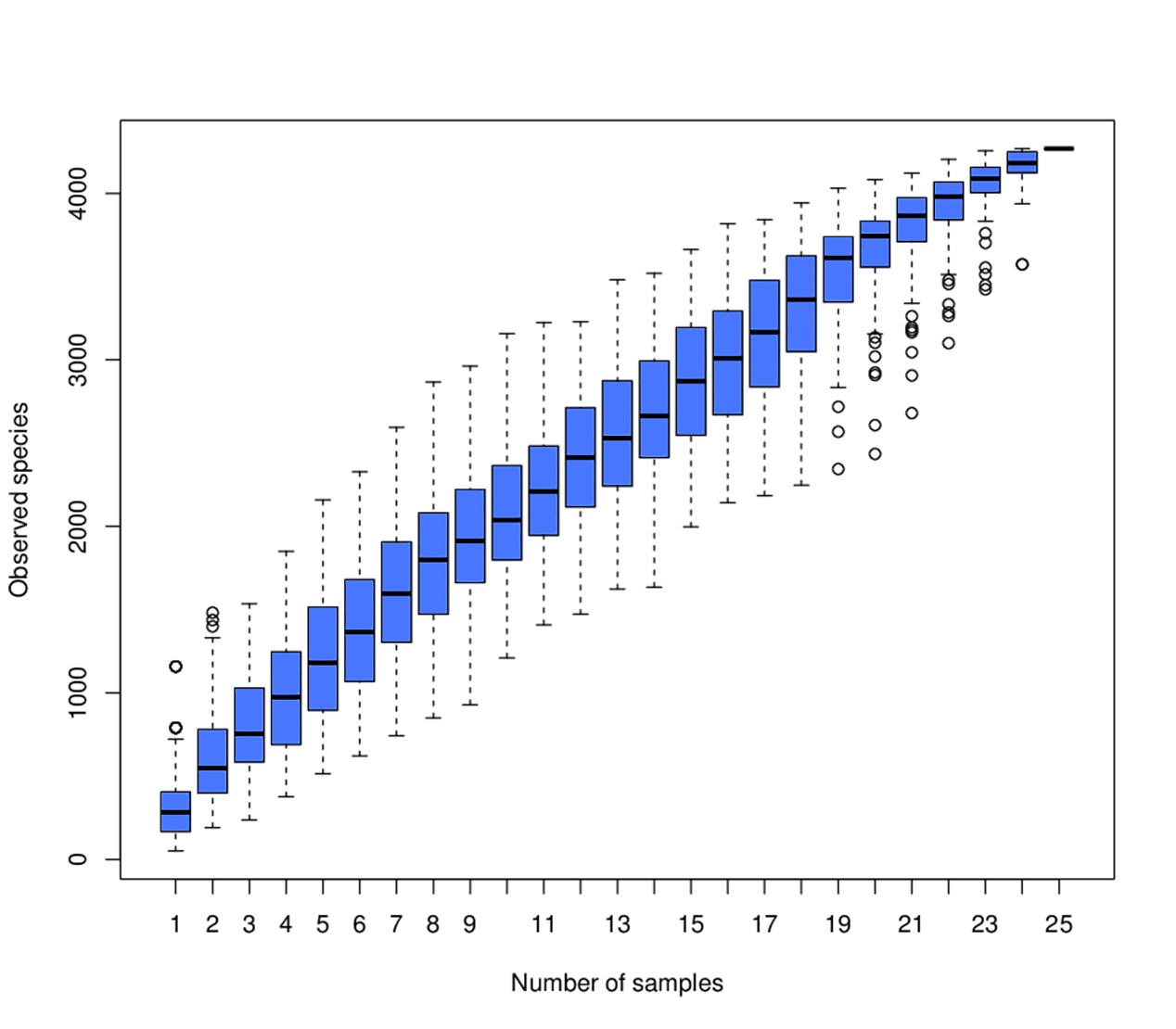


**Figure S3: Plot Species Accumulation curves**

Figure S3: the abscissa represents the sample size; the ordinate represents the number of OTUs after sampling, which reflects the rate at which new OTUs (new species) appear under continuous sampling.
